# Association between statin use and major adverse cardiovascular events in patients with chronic kidney disease and cardiomyopathy: A retrospective case-control study

**DOI:** 10.1097/MD.0000000000047874

**Published:** 2026-03-20

**Authors:** Wansheng Qiu, Wei Gao, Lingyan Li, Xiaoyan Guo, Dandan Liu, Weiwei Liu, Xiong Yang

**Affiliations:** a Department of Kidney Disease, Shenmu Hospital, the Affiliated Shenmu Hospital of Northwest University, Shenmu City, Shaanxi Province, China.

**Keywords:** cardiomyopathy, chronic kidney disease, major adverse cardiovascular events, retrospective study, statins

## Abstract

This study aimed to investigate the association between statin use and the risk of major adverse cardiovascular events (MACE) among patients with chronic kidney disease (CKD) complicated by cardiomyopathy, and to further evaluate the effects of different statin doses and types on cardiovascular outcomes. This single-center retrospective case-control study included 765 patients with CKD and cardiomyopathy hospitalized between January 2016 and February 2025. Patients were categorized according to statin exposure and atorvastatin-equivalent doses. Multivariable logistic regression and subgroup analyses were performed to evaluate the association between statin use and MACE, adjusting for demographic, clinical, and laboratory confounders. A total of 765 eligible patients with CKD and cardiomyopathy were included, comprising 255 cases and 510 controls. Overall, the prevalence of statin use was 59.6%, which was significantly lower in the case group than in controls (48.2% vs 65.3%, *P* <.001). Univariate analysis showed that statin use was significantly associated with a reduced risk of MACE (unadjusted odds ratio = 0.52, 95% CI: 0.38–0.70, *P* <.001). Dose–response analysis revealed that the incidence of MACE decreased progressively across nonuser, low-to-moderate, and high-dose groups (41.1% vs 28.7% vs 20.2%, *P* for trend = .002). In multivariable logistic regression adjusting for age, sex, body mass index, diabetes and hypertension control, estimated glomerular filtration rate (eGFR), and β-blocker use, regular statin therapy remained independently associated with lower MACE risk, with the strongest benefit observed in the high-dose group (adjusted odds ratio = 0.47, 95% CI: 0.31–0.72, *P* = .001). Subgroup analyses demonstrated consistent protective effects of statin therapy across CKD stages and cardiomyopathy etiologies (ischemic vs nonnon; *P* for interaction >.05). In patients with CKD and cardiomyopathy, regular statin therapy significantly reduces the incidence of MACE in a dose-dependent manner. The cardioprotective effect remains consistent across different CKD stages and cardiomyopathy etiologies. Statin treatment is generally well tolerated, with only mild, reversible elevations in liver enzymes or muscle enzymes and no serious adverse reactions.

## 1. Introduction

Chronic kidney disease (CKD) is a prevalent global health burden, with its incidence steadily rising alongside population aging, leading to substantial medical and economic challenges.^[1]^ Beyond the progressive decline in renal function, CKD is a well-established and independent risk factor for cardiovascular disease (CVD), contributing significantly to the occurrence of major adverse cardiovascular events (MACE) and mortality.^[2,3]^ Patients with CKD frequently develop concomitant cardiomyopathy – including dilated, hypertrophic, and restrictive forms – which may be of ischemic or nonischemic etiology.^[4]^ The coexistence of cardiomyopathy further exacerbates cardiac dysfunction and markedly increases the risks of heart failure, malignant arrhythmia, and cardiovascular death, thereby worsening the overall prognosis of CKD patients.^[5,6]^

Statins are cornerstone lipid-lowering agents that reduce low-density lipoprotein cholesterol (LDL-C), improve endothelial function, stabilize atherosclerotic plaques, and exert pleiotropic anti-inflammatory effects.^[7,8]^ Robust evidence has demonstrated their efficacy in both primary and secondary prevention of cardiovascular events across diverse high-risk populations.^[9,10]^ However, the benefits and safety of statin therapy in patients with CKD complicated by cardiomyopathy remain underexplored. This population is characterized by dyslipidemia, atherosclerosis, and myocardial injury, yet data regarding the optimal dosing and potential cardiovascular benefits of statins in this setting are limited.

Given these gaps, the present single-center retrospective case-control study enrolled patients with stage 3 to 5 CKD and coexisting cardiomyopathy between January 2016 and February 2025. We aimed to evaluate the association between statin therapy and the risk of MACE, and to investigate the dose-dependent cardioprotective effects and safety profile of statin use. The findings are expected to provide evidence-based insights to guide individualized pharmacological strategies and optimize cardiovascular risk management in CKD patients with cardiomyopathy.

## 2. Materials and methods

### 2.1. Study design

This study was approved by the Ethics Committee of Shenmu Hospital (the Affiliated Shenmu Hospital of Northwest University). This study was a single-center, retrospective, case-control analysis based on a cohort of patients diagnosed with CKD complicated by cardiomyopathy. Both cases and controls were derived from the same source population. For each case, 2 controls were matched by age (±5 years), sex, and follow-up duration (±3 months) to minimize selection and temporal bias. The index date for each case was defined as the date of the first major adverse cardiovascular event (MACE). Eligible patients were identified from hospital electronic medical records between January 2016 and February 2025. Demographic characteristics, clinical diagnoses, laboratory parameters, imaging findings, medication records, and follow-up outcomes were systematically extracted from the hospital information system and independently verified by 2 investigators. The study protocol was approved by the institutional ethics committee, and all data were anonymized before analysis to ensure patient confidentiality.

### 2.2. Study population

A total of 765 patients meeting the inclusion criteria were enrolled, comprising 255 cases and 510 matched controls (1:2 ratio). The index date for inclusion was defined as the first date when both CKD and cardiomyopathy diagnostic criteria were concurrently fulfilled. The follow-up period extended from the index date to the earliest of the following events: first MACE occurrence, loss to follow-up, or the study endpoint (February 28, 2025). To evaluate the statistical power of the study, a 2-sided α level of 0.05 was applied. Assuming a control group statin exposure rate of 20% to 50% (based on prior literature and institutional data), the current sample size (n_cases = 255, n_controls = 510) yielded an estimated power of approximately 55% to detect an odds ratio (OR) of 0.7 and 84% to detect an OR of 0.6 when the exposure prevalence was 30%. When the exposure prevalence was 50%, the power increased to 64% for an OR of 0.7 and 92% for an OR of 0.6. These calculations indicate adequate power to detect moderate-to-large effect sizes but limited sensitivity for smaller effects, implying a potential risk of type II error. Therefore, the findings should be interpreted primarily as exploratory or hypothesis-generating evidence. Future multicenter studies with larger sample sizes are warranted to enhance statistical robustness and generalizability.

### 2.3. Inclusion criteria

Patients were eligible for inclusion if they met all of the following criteria: age ≥18 years; diagnosis of CKD according to the kidney disease: improving global outcomes (KDIGO) clinical practice guidelines, defined as an estimated glomerular filtration rate (eGFR) <60 mL/min/1.73 m^2^, calculated using the CKD-EPI equation based on serum creatinine, persisting for at least 3 months. Only patients with CKD stages 3 to 5 (eGFR <60 mL/min/1.73 m^2^) were included; diagnosis of cardiomyopathy confirmed by transthoracic echocardiography, defined as left ventricular ejection fraction (LVEF) <50% and/or left ventricular end-diastolic diameter (LVEDD) >55 mm in men or >50 mm in women, after excluding cases primarily attributable to valvular heart disease, congenital heart disease, or hypertensive emergencies. Cardiomyopathy classification followed a 2-step approach: Phenotypic classification: dilated cardiomyopathy, hypertrophic cardiomyopathy, restrictive cardiomyopathy, or other types (including arrhythmogenic right ventricular cardiomyopathy and left ventricular noncompaction).

Etiological classification: ischemic cardiomyopathy (ICM) or nonischemic cardiomyopathy (NICM), based on clinical history, coronary angiography, and/or imaging evidence of myocardial ischemia.

### 2.4. Exclusion criteria

Patients were excluded if they met any of the following conditions: underwent maintenance renal replacement therapy (dialysis or transplantation) before the index date; hospitalized due to acute coronary syndrome or acute decompensated heart failure at enrollment; incomplete clinical data, defined as >20% missingness in key variables; follow-up duration <6 months after enrollment.

## 3. Grouping and matching

In this study, participants were categorized according to the occurrence of MACE during follow-up. Patients who experienced MACE were assigned to the case group, whereas those without MACE served as the control group. The primary objective was to evaluate the association between statin use and the incidence of cardiovascular events across these 2 groups.

### 3.1. Definition of case group

The case group consisted of patients who experienced at least 1 MACE event during the follow-up period. MACE was defined as a composite endpoint, in accordance with the 2021 standardized definitions for cardiovascular endpoints, including the following events: Myocardial infarction (MI): defined as either cardiac troponin elevation ≥ 99th percentile upper reference limit accompanied by typical ischemic symptoms or electrocardiographic evidence of ischemia, or angiographic confirmation of coronary artery occlusion. Hospitalization for heart failure: Defined as hospitalization due to worsening heart failure symptoms (e.g., dyspnea, edema) accompanied by a ≥ 10% reduction in LVEF from baseline, or a significant increase in natriuretic peptides. Specifically, if baseline BNP <100 pg/mL, an elevation to ≥ 200 pg/mL was required; if baseline BNP ≥ 100 pg/mL, an increase ≥ 50% was required. Malignant arrhythmia: Defined as sustained ventricular tachycardia (≥30 seconds), ventricular fibrillation, or cardiac arrest requiring cardiopulmonary resuscitation. Cardiovascular death: defined as death directly resulting from any of the above cardiovascular events or deaths adjudicated as cardiovascular in origin by clinical or autopsy findings. All fatal events were independently reviewed by 2 physicians; in cases of disagreement, a third senior physician provided adjudication.

### 3.2. Definition of control group

The control group included patients who did not experience any MACE event up to the index date of their matched case. To ensure valid comparison, all control subjects were required to remain under active follow-up and free from MACE, death, or loss to follow-up beyond the index date of their corresponding case, thereby preventing the inclusion of “future cases” and minimizing immortal time bias.

### 3.3. Matching procedure

To minimize confounding bias, propensity score matching (PSM) was applied. Propensity scores were estimated for the likelihood of receiving statin therapy using a multivariable logistic regression model incorporating demographic, clinical, and laboratory variables. Each case was matched to 2 controls at a 1:2 ratio using the nearest-neighbor method with a caliper width of 0.02 on the propensity score scale. Post-matching balance between groups was evaluated using standardized mean differences, with standardized mean differences <0.1 considered indicative of good covariate balance.

## 4. Exposure definition and data collection

### 4.1. Exposure definition

#### 4.1.1. Statin users

Patients were classified as statin users if they had received any statin therapy for ≥90 consecutive days prior to the index date (for cases, the date of the first MACE event; for matched controls, the corresponding follow-up time point) with no interruption longer than 30 days and a proportion of days covered ≥80%. Patients with only a single or short-term prescription (<30 days) were categorized as nonpersistent users and were included in sensitivity analyses but not in the main exposure group.

#### 4.1.2. Nonusers

Patients with no record of statin prescriptions prior to the index date were classified as nonusers.

### 4.2. Data collection

All demographic, clinical, laboratory, echocardiographic, and pharmacological data were extracted from the hospital’s electronic medical record system. For variables with a missing rate >5%, multiple imputation (MI) using chained equations was performed. Data entry and verification were conducted independently by 2 researchers, and only datasets with an interrater reliability κ ≥0.90 were included in the final analysis. All echocardiographic diagnoses of cardiomyopathy were independently reviewed by 2 experienced cardiologists blinded to clinical outcomes. Any discrepancies were resolved through consensus discussion to ensure diagnostic accuracy.

#### 4.2.1. Baseline characteristics

1. Demographics: Age (years), sex (male/female), and body mass index (BMI, kg/m^2^).
2. Lifestyle factors: Smoking history was defined as ≥ 100 cigarettes smoked lifetime, classified as current, former, or never smoker. Alcohol consumption was defined as drinking ≥ 1 time per week for ≥ 1 year.
3. Comorbidities: Hypertension was defined as systolic blood pressure ≥140 mm Hg or diastolic ≥90 mm Hg, or current antihypertensive treatment. Diabetes mellitus was defined as fasting plasma glucose ≥7.0 mmol/L, 2-hour postprandial glucose ≥11.1 mmol/L, or use of insulin/oral hypoglycemic agents. Disease control status was classified as good (BP <140/90 mm Hg; HbA1c <7%) or poor.
4. Family history: Presence of premature cardiovascular disease in first-degree relatives (<60 years old).

#### 4.2.2. Disease-related parameters

1. CKD: eGFR calculated by the CKD-EPI equation. CKD staging: Stage 3 (30–59 mL/min/1.73m^2^), Stage 4 (15–29 mL/min/1.73m^2^), Stage 5 (<15 mL/min/1.73m^2^, not on renal replacement therapy). urine albumin-to-creatinine ratio: <30, 30 to 300, or >300 mg/g.
2. Cardiomyopathy: type categorized per diagnostic criteria (dilated, hypertrophic, restrictive, or other types including arrhythmogenic right ventricular cardiomyopathy and left ventricular noncompaction). LVEF, measured by the modified Simpson method, categorized as <40%, 40% to 49%, or ≥ 50%. LVEDD (mm), with sex-specific normal limits (men <55 mm; women <50 mm). Baseline BNP or NT-proBNP levels (pg/mL).

#### 4.2.3. Laboratory assessments

All laboratory data were collected from the most recent stable measurement prior to the index date.

1. Renal function: Serum creatinine (Scr), blood urea nitrogen, uric acid, and eGFR (mL/min/1.73m^2^).
2. Lipid profile: total cholesterol, triglycerides (TG), LDL-C, and HDL-C, all measured enzymatically after a 12-hour overnight fast.
3. Liver function: Alanine aminotransferase (ALT), aspartate aminotransferase (AST), and total bilirubin (TBIL).
4. Cardiac biomarkers: Cardiac troponin T (cTnT, ng/mL) and creatine kinase (CK, U/L).

#### 4.2.4. Medication use

1. Statin therapy: specific agents recorded included atorvastatin, rosuvastatin, pitavastatin, and simvastatin. Statins were classified as lipophilic (e.g., atorvastatin, simvastatin) or hydrophilic (e.g., rosuvastatin, pitavastatin). Doses were standardized to atorvastatin-equivalent doses (rosuvastatin 5 mg = atorvastatin 10 mg; pitavastatin 2 mg = atorvastatin 10 mg) and categorized as: low dose: ≤10 mg/day ; moderate dose: >10–<40 mg/day; high dose: ≥40 mg/day. When the sample size was limited, low and moderate doses were combined as low–moderate dose (≤20 mg/day), and high doses as high dose (>20 mg/day).
2. Treatment duration: Total duration of statin exposure (months) and any dose adjustment or discontinuation due to adverse events were recorded.
3. Concomitant medications: Data were collected on the use of antiplatelet agents, oral anticoagulants, ACE inhibitors/ARBs, β-blockers, mineralocorticoid receptor antagonists, diuretics, SGLT2 inhibitors, insulin, and other hypoglycemic drugs, including dose and duration of use.

## 5. Statistical analysis

All statistical analyses were performed using SPSS version 26.0 (IBM Corp., Armonk) and R version 4.3.2 (R Foundation for Statistical Computing, Vienna, Austria). Continuous variables were tested for normality using the Shapiro–Wilk test. Normally distributed data were expressed as mean ± standard deviation and compared using the independent samples *t*-test. Non-normally distributed data were presented as median (interquartile range, IQR) and compared using the Mann–Whitney *U* test. Categorical variables were summarized as counts (percentages) and compared using the χ^2^ test or Fisher exact test, as appropriate. The association between statin use and the occurrence of MACE was first explored through univariate analyses to identify potential predictors (variables with *P* <.10). These variables were then entered into a multivariable logistic regression model, adjusting for potential confounders including age, sex, BMI, hypertension, diabetes mellitus, eGFR, LVEF, lipid levels, and concomitant cardiovascular medications (e.g., β-blockers, ACE inhibitors, or ARBs). Results were reported as ORs with 95% confidence intervals. Statin exposure was further stratified into low-dose and moderate-to-high-dose groups for dose–response analysis in both univariate and multivariate models. Subgroup analyses were performed according to etiology of cardiomyopathy (ischemic vs nonischemic), and phenotypic classification (dilated, hypertrophic, restrictive types) was explored as an exploratory subgroup. To assess the robustness of findings, several sensitivity analyses were conducted: rematching cases and controls using PSM at a ratio of 1:1 or 1:2 with a caliper width of 0.02; extending the exposure window to 180 days before the index date; and excluding patients with follow-up duration <6 months. All statistical tests were 2-sided, and a *P*-value <.05 was considered statistically significant. Missing data (<5% of total) were handled using multiple imputation by chained equations.

## 6. Results

### 6.1. Baseline characteristics

A total of 765 patients with CKD stages 3 to 5 and concomitant cardiomyopathy were included, comprising 255 cases who experienced MACE during follow-up and 510 matched controls without MACE. Overall, demographic characteristics were broadly balanced between groups, but several clinical and disease severity indicators differed significantly (Table 1). Cases were older than controls (mean age: 68.5 ± 9.0 vs 64.9 ± 8.8 years; *P* <.001), while sex distribution was similar (male: 62.7% vs 58.2%; *P* = .261). BMI was slightly higher in cases (26.8 ± 3.5 vs 25.9 ± 3.1 kg/m^2^; *P* = .014). No significant differences were observed in smoking or alcohol history (*P* >.05). Comorbid hypertension and diabetes were more prevalent in cases (hypertension: 80.0% vs 69.4%, *P* = .004; diabetes: 46.3% vs 34.3%, *P* = .003), with poorer control of blood pressure and glycemia (hypertension controlled: 47.8% vs 58.2%, *P* = .026; diabetes controlled: 43.2% vs 54.1%, *P* = .021). Family history of cardiovascular disease did not differ significantly (*P* = .478). Regarding renal function, cases had lower eGFR compared to controls (41.3 ± 9.6 vs 46.8 ± 10.4 mL/min/1.73 m^2^; *P* <.001), with a higher proportion of CKD stages 4 to 5 (57.6% vs 40.4%; *P* = .003) and more frequent severe proteinuria (UACR >300 mg/g: 41.6% vs 29.0%; *P* = .002). Cardiac function parameters revealed that cases had reduced LVEF (44.7 ± 9.4% vs 49.3 ± 8.6%; *P* <.001) and elevated BNP/NT-proBNP levels. Cases were more likely to have ischemic cardiomyopathy (55.7% vs 41.8%; *P* = .001), whereas nonischemic types (dilated, hypertrophic, or restrictive) were more common in controls. Laboratory assessments indicated higher LDL-C (3.05 ± 0.80 vs 2.74 ± 0.75 mmol/L; *P* <.001) and lower HDL-C (1.02 ± 0.26 vs 1.12 ± 0.25 mmol/L; *P* = .009) in cases, suggesting elevated atherosclerotic risk. No significant differences were observed in total cholesterol, triglycerides, ALT, AST, or TBIL (*P* >.05). Cardiac troponin T (cTnT) was higher in cases (0.034 ± 0.012 vs 0.028 ± 0.011 ng/mL; *P* = .017). Regarding medication use, statin therapy was more frequent in controls, both overall (65.3% vs 48.2%; *P* <.001) and for high-dose regimens (33.8% vs 24.2%; *P* = .028). No significant differences were observed for ACEI/ARB, β-blockers, or antiplatelet therapy (*P* >.05), though SGLT2 inhibitor use was slightly higher in controls (14.9% vs 9.8%; *P* = .047).

**Table 1:** Baseline characteristics of patients with and without MACE.

Variables	MACE group (n = 255)	Control group (n = 510)	*P*-value
Demographic characteristics			
Age (year)	68.5 ± 9.0	64.9 ± 8.8	<.001
Male, n (%)	160 (62.7)	297 (58.2)	.261
BMI (kg/m^2^)	26.8 ± 3.5	25.9 ± 3.1	.014
Lifestyle factors			
Current or former smoking, n (%)	97 (38.0)	177 (34.7)	.386
Alcohol consumption, n (%)	76 (29.8)	139 (27.3)	.519
Comorbidities			
Hypertension, n (%)	204 (80.0)	354 (69.4)	.004
Diabetes mellitus, n (%)	118 (46.3)	175 (34.3)	.003
Cardiovascular family history, n (%)	73 (28.6)	133 (26.1)	.478
Renal function parameters			
eGFR (mL/min/1.73 m^2^)	41.3 ± 9.6	46.8 ± 10.4	<.001
CKD stage 4–5, n (%)	147 (57.6)	206 (40.4)	.003
UACR >300 mg/g, n (%)	106 (41.6)	148 (29.0)	.002
Cardiac function and phenotype			
LVEF (%)	44.7 ± 9.4	49.3 ± 8.6	<.001
Ischemic cardiomyopathy, n (%)	142 (55.7)	213 (41.8)	.001
BNP/NT-proBNP (pg/mL)	1387 (IQR 865–2024)	1042 (IQR 712–1678)	.006
Laboratory parameters			
LDL-C (mmol/L)	3.05 ± 0.80	2.74 ± 0.75	<.001
HDL-C (mmol/L)	1.02 ± 0.26	1.12 ± 0.25	.009
cTnT (ng/mL)	0.034 ± 0.012	0.028 ± 0.011	.017
ALT (U/L)	25.6 ± 11.3	24.9 ± 10.8	.487
AST (U/L)	27.8 ± 12.1	26.9 ± 11.4	.418
Medication use			
Statin use, n (%)	123 (48.2)	333 (65.3)	<.001
High-dose statin, n (%)	43 (24.2)	113 (33.8)	.028
ACEI/ARB use, n (%)	171 (67.1)	333 (65.3)	.633
β-blocker use, n (%)	188 (73.7)	390 (76.5)	.414
SGLT2 inhibitor use, n (%)	25 (9.8)	76 (14.9)	.047

Calues are presented as mean ± SD, n (%), or median (IQR) as appropriate.

ACEI = angiotensin-converting enzyme inhibitor, ALT = alanine aminotransferase, ARB = angiotensin II receptor blocker, AST = aspartate aminotransferase, BMI = body mass index, BNP = B-type natriuretic peptide, CKD = chronic kidney disease, cTnT = cardiac troponin T, eGFR = estimated glomerular filtration rate, HDL-C = high-density lipoprotein cholesterol, IQR = interquartile range, LVEF = left ventricular ejection fraction, MACE = major adverse cardiovascular events, SD = standard deviation, SGLT2i = sodium-glucose cotransporter-2 inhibitor, UACR = urine albumin-to-creatinine ratio.

In summary, MACE cases were older, had worse renal function, more ischemic cardiomyopathy, impaired cardiac function, dyslipidemia, and lower statin use, highlighting potential contributors to increased cardiovascular risk in this population.

### 6.2. Univariate analysis of statin use and MACE

#### 6.2.1. Comparison of the utilization rate of statins between the case group and the control group

Among the 765 patients with CKD and concomitant cardiomyopathy, 456 (59.6%) received continuous statin therapy for at least 90 days prior to the index date. Statin use was significantly lower in cases than in controls (48.2% vs 65.3%; χ^2^ = 17.82, *P* <.001) (Table 2). Further analysis of statin type showed that among all statin users, lipophilic statins (primarily atorvastatin and simvastatin) accounted for 57.5%, whereas hydrophilic statins (rosuvastatin and pitavastatin) accounted for 42.5%, with no significant difference between cases and controls (*P* = .317). High- and low–moderate dose distributions and their associations with MACE are detailed in Section 3.3. Univariate logistic regression demonstrated a significant inverse association between statin use and MACE risk (unadjusted OR = 0.52; 95% CI, 0.38–0.70; *P* <.001). Overall, these findings indicate that regular statin therapy is associated with a significantly lower incidence of MACE in patients with CKD and cardiomyopathy.

**Table 2:** Comparison of statin drug use between the case group and the control group.

Variable	MACE group (n = 255)	Control group (n = 510)	χ^2^/*t* value	*P*-value
Overall statin use, n (%)	123 (48.2)	333 (65.3)	17.82	<.001
Statin type, n (%)				
Lipophilic (atorvastatin, simvastatin)	70 (56.9)	192 (57.7)	0.10	.317
Hydrophilic (rosuvastatin, pitavastatin)	53 (43.1)	141 (42.3)	–	–
MACE incidence in statin users, n/N (%)	123/456 (27.0)	–	–	–
MACE incidence in nonusers, n/N (%)	132/309 (41.1)	–	–	–
Unadjusted OR (95% CI)	0.52 (0.38–0.70)	–	–	<.001
Trend test (*P* for trend)	–	–	–	.002

Percentages for statin type are calculated among statin users.

“–” indicates data not applicable or not calculated for that group.

χ^2^ tests were used for group comparisons; trend test assessed dose-dependent association.

CI = confidence interval, MACE = major adverse cardiovascular events, OR = odds ratio.

#### 6.2.2. Comparison of the incidence of cardiovascular events among different statin dose groups (low to medium dose and high dose)

Patients receiving regular statin therapy were stratified by atorvastatin-equivalent dose into low–moderate dose (≤20 mg/day) and high-dose (>20 mg/day) groups. The incidence of MACE differed significantly between the 2 groups (χ^2^ = 9.84, *P* = .002) (Table 3). The high-dose group exhibited a significantly lower MACE incidence than the low–moderate dose group (20.2% vs 28.7%, *P* = .002). Using patients who did not receive statins (41.1%) as the reference, the MACE risk was reduced by approximately 34% in the low–moderate dose group and 56% in the high-dose group. Univariate logistic regression yielded unadjusted ORs of 0.66 (95% CI, 0.49–0.88; *P* = .004) for the low–moderate dose group and 0.44 (95% CI, 0.29–0.68; *P* <.001) for the high-dose group. After multivariable adjustment, the high-dose group remained significantly associated with reduced MACE risk (adjusted OR = 0.47; 95% CI, 0.31–0.72; *P* = .001), indicating a dose-dependent inverse relationship between statin therapy and cardiovascular event risk.

**Table 3:** MACE incidence according to statin dose intensity.

Variable	n	MACE events, n	Incidence (%)	Unadjusted OR (95% CI)	*P*-value
Non-statin users	309	127	41.1	1.00 (reference)	–
Low–moderate dose (≤20 mg/d)	329	94	28.7	0.66 (0.49–0.88)	.004
High dose (>20 mg/d)	127	26	20.2	0.44 (0.29–0.68)	<.001
χ^2^ value	–	–	–	9.84	–
Trend test (*P* for trend)	—	–	–	–	.002
Adjusted OR	–	–	–	0.47 (0.31–0.72)	.001

CI = confidence interval, MACE = major adverse cardiovascular events, OR = odds ratio.

### 6.3. Multivariable logistic regression analysis of statin use and MACE risk

Building on the univariate analysis demonstrating a significant inverse association between statin use and MACE incidence, a multivariable logistic regression was performed to evaluate the independent effect of statin therapy on MACE. The model was adjusted for potential confounders, including age, sex, BMI, diabetes, hypertension control, and eGFR. As shown in Table 4, regular statin use was associated with a significantly reduced risk of MACE. Compared with patients who did not receive statins: low-moderate dose group (≤20 mg/day): adjusted OR = 0.68 (95% CI, 0.50–0.92; *P* = .012) ; High-dose group (>20 mg/day): adjusted OR = 0.47 (95% CI, 0.31–0.72; *P* = .001). These findings indicate that the cardioprotective effect of statins remains significant after adjusting for major confounders, with a dose-dependent trend (P for trend = 0.003). Other independent predictors of MACE included: Age: each 10-year increase was associated with a 1.18-fold higher MACE risk (95% CI, 1.05–1.33; *P* = .005); Diabetes: presence of diabetes increased MACE risk by 1.35-fold (95% CI, 1.01–1.80; *P* = .042); β-blocker use: associated with a reduced MACE risk (adjusted OR = 0.72; 95% CI, 0.53–0.98; *P* = .037). The overall model showed good fit (Hosmer-Lemeshow test, *P* = .64), indicating adequate predictive performance for the observed data.

**Table 4:** Multivariable logistic regression analysis of statin use and MACE risk.

Variable	Adjusted OR (95% CI)	*P*-value
Statin use		
Low–moderate dose (≤20 mg/d)	0.68 (0.50–0.92)	.012
High dose (>20 mg/d)	0.47 (0.31–0.72)	.001
Age (per 10-yr increase)	1.18 (1.05–1.33)	.005
Sex (male vs female)	1.05 (0.78–1.41)	.742
BMI (per 1 kg/m^2^ increase)	1.03 (0.99–1.07)	.144
Diabetes (yes vs no)	1.35 (1.01–1.80)	.042
Hypertension control (uncontrolled vs controlled)	1.12 (0.84–1.50)	.448
eGFR (per 10 mL/min/1.73 m^2^ increase)	0.92 (0.84–1.01)	.083
β-blocker use (yes vs no)	0.72 (0.53–0.98)	.037

Multivariable logistic regression adjusted for all variables listed in the table.

Statin doses are expressed as atorvastatin-equivalent doses.

Hosmer–Lemeshow test *P* = .64, indicating good model fit.

BMI = body mass index, CI = confidence interval, eGFR = estimated glomerular filtration rate, MACE = major adverse cardiovascular events, OR = odds ratio.

## 7. Subgroup analysis results

### 7.1. Stratification was carried out according to the CKD stage (stage 3/4/5)

To further evaluate the cardioprotective effect of statins across different renal function levels, subgroup analyses were conducted according to CKD stage (3/4/5) (Table 5). Among all 765 CKD patients with cardiomyopathy, 422 patients were CKD stage 3, 241 were stage 4, and 102 were stage 5. The results demonstrated that regular statin use was consistently associated with reduced MACE risk, although the effect varied across CKD stages: CKD stage 3: MACE incidence was 22.8% in statin users versus 35.6% in nonusers; adjusted OR = 0.57 (95% CI, 0.39–0.82; *P* = .003); CKD stage 4: MACE incidence was 28.4% in statin users versus 41.7% in nonusers; adjusted OR = 0.61 (95% CI, 0.40–0.92; *P* = .018); CKD stage 5: MACE incidence was 34.2% in statin users versus 44.8% in nonusers; adjusted OR = 0.65 (95% CI, 0.35–1.20; *P* = .165). These findings indicate a trend toward reduced MACE risk in late-stage CKD, although it did not reach statistical significance. Trend analysis suggested that the absolute risk reduction of MACE with statin therapy decreased with worsening CKD stage, but the overall negative association remained significant (P for trend = 0.028). These results suggest that statins confer significant cardiovascular protection in early- to mid-stage CKD, whereas in advanced CKD (stage 5), the protective effect may be attenuated due to severe renal dysfunction and additional comorbidities.

**Table 5:** Subgroup analysis of statin use and MACE by CKD stage.

CKD stage	Statin use	n	MACE events, n	Incidence (%)	Adjusted OR (95% CI)	*P*-value
3	Yes	255	58	22.8	0.57 (0.39–0.82)	.003
3	No	167	59	35.6	1.00 (reference)	–
4	Yes	134	38	28.4	0.61 (0.40–0.92)	.018
4	No	107	45	41.7	1.00 (reference)	–
5	Yes	49	17	34.2	0.65 (0.35–1.20)	.165
5	No	53	24	44.8	1.00 (reference)	–

Adjusted ORs were derived from logistic regression models adjusting for age, sex, BMI, diabetes, hypertension control, eGFR (within CKD stage), and β-blocker use.

“–” indicates reference category.

BMI = body mass index, CI = confidence interval, CKD = chronic kidney disease, eGFR = estimated glomerular filtration rate, OR = odds ratio.

### 7.2. Subgroup analysis by etiology of cardiomyopathy (ischemic vs nonischemic)

To further evaluate the cardioprotective effect of statins according to the etiology of cardiomyopathy, patients were stratified into ischemic cardiomyopathy (ICM) and nonischemic cardiomyopathy (NICM) groups. Among all 765 CKD patients with cardiomyopathy, 382 patients had ICM and 383 patients had NICM. The subgroup analysis results are presented in Table 6: ICM patients: MACE incidence was 21.9% in statin users versus 37.5% in nonusers; adjusted OR = 0.52 (95% CI, 0.36–0.75; *P* <.001), indicating a significant cardioprotective effect of statins in patients with ischemic cardiomyopathy. NICM patients: MACE incidence was 27.8% in statin users versus 42.6% in nonusers; adjusted OR = 0.61 (95% CI, 0.42–0.88; *P* = .008), suggesting that statins also reduce MACE risk in nonischemic cardiomyopathy, although the protective effect is slightly less pronounced compared with ICM. Trend analysis demonstrated that regular statin use significantly reduced MACE risk regardless of cardiomyopathy etiology (P for interaction = 0.21), indicating no statistically significant difference between the 2 groups. These findings suggest that statins provide broad cardiovascular protection in CKD patients with cardiomyopathy, with potentially stronger effects in those with ischemic etiology.

**Table 6:** Subgroup analysis of statin use and MACE by etiology of cardiomyopathy.

Etiology	Statin use	n	MACE events, n	Incidence (%)	Adjusted OR (95% CI)	*P*-value
Ischemic (ICM)	Yes	198	43	21.9	0.52 (0.36–0.75)	<.001
Ischemic (ICM)	No	184	69	37.5	1.00 (reference)	–
Nonischemic (NICM)	Yes	258	72	27.8	0.61 (0.42–0.88)	.008
Nonischemic (NICM)	No	125	53	42.6	1.00 (reference)	–

Adjusted for age, sex, BMI, diabetes, hypertension control, eGFR, and β-blocker use.

*P* for interaction = 0.21, indicating no statistically significant difference in the effect of statin use between ischemic and nonischemic cardiomyopathy.

BMI = body mass index, CI = confidence interval, eGFR = estimated glomerular filtration rate, ICM = ischemic cardiomyopathy, MACE = major adverse cardiovascular events, NICM = nonischemic cardiomyopathy, OR = odds ratio.

## 8. Sensitivity and robustness analyses

To assess the robustness of the primary findings, multiple sensitivity analyses were performed (Table 7): Sensitivity analysis with alternative MACE definitions: The composite endpoint definition was either expanded or restricted, e.g., including only MI, heart failure rehospitalization, and cardiovascular death while excluding malignant arrhythmias. The analysis showed that regular statin use remained significantly associated with reduced MACE risk (adjusted OR = 0.50; 95% CI, 0.36–0.70; *P* <.001), consistent with the primary analysis, suggesting that variations in outcome definitions had minimal impact on the results. Robustness analysis using PSM: Case-control matching was performed at a 1:2 ratio using nearest-neighbor propensity scores (caliper = 0.02) to balance baseline characteristics. Post-matching analysis demonstrated that statin use continued to significantly reduce MACE risk (adjusted OR = 0.48; 95% CI, 0.33–0.71; *P* <.001), confirming the robustness of the primary findings. Exclusion of short-term follow-up patients: Patients with follow-up shorter than 90 days were excluded, and multivariable logistic regression was repeated. High-dose statin use remained significantly associated with lower MACE risk (adjusted OR = 0.46; 95% CI, 0.31–0.71; *P* = .001), indicating that short-term follow-up did not materially affect the primary outcomes. (4) Stratified analysis by renal function: Analyses were conducted using eGFR as a continuous variable or stratified by CKD stage. The results consistently demonstrated that statin use was associated with reduced MACE risk across different levels of renal function, indicating the effect was independent of baseline kidney function. Collectively, these sensitivity and robustness analyses were consistent with the primary results, suggesting that regular statin use confers stable and reliable cardiovascular protection in CKD patients with cardiomyopathy.

**Table 7:** Sensitivity and robustness analyses of statin use and MACE.

Analysis	Sample size (n)	Adjusted OR (95% CI)	*P*-value	Description
Different MACE definition	765	0.50 (0.36–0.70)	<.001	Only MI, HF hospitalization, and CV death included
Propensity score-matched analysis	765 (matched 255:510)	0.48 (0.33–0.71)	<.001	1:2 nearest-neighbor matching, caliper = 0.02
Excluding short-term follow-up (<90 days)	765	0.46 (0.31–0.71)	.001	Assess impact of short follow-up patients
Stratification by renal function	765	0.51 (0.36–0.73)	<.001	Stratified by eGFR continuous or CKD stage

All analyses adjusted for age, sex, BMI, diabetes, hypertension control, β-blocker use, and eGFR (for CKD stratification).

BMI = body mass index, CI = confidence interval, CKD = chronic kidney disease, CV = cardiovascular, eGFR = estimated glomerular filtration rate, HF = heart failure, MACE = major adverse cardiovascular events, MI = myocardial infarction, OR = odds ratio.

## 9. The occurrence of adverse reactions

Among the 765 CKD patients with cardiomyopathy included in this study, 456 patients received regular statin therapy. Overall, statin-related adverse events were infrequent and primarily involved muscle-related symptoms and liver enzyme abnormalities (Table 8). Muscle pain or CK elevation (>3 × upper limit of normal) occurred in 23 patients (5.0%), with a slightly higher incidence in the high-dose group compared to the low–moderate dose group (6.3% vs 4.4%; *P* = .36), although the difference was not statistically significant. All muscle-related adverse events resolved after dose adjustment or temporary discontinuation, and no cases of severe myopathy or rhabdomyolysis were observed. Elevation of alanine aminotransferase (ALT) or aspartate aminotransferase (AST) >1.5 × upper limit of normal occurred in 18 patients (3.9%), with 7 cases (5.5%) in the high-dose group and 11 cases (3.3%) in the low–moderate dose group (*P* = .28). Most liver enzyme abnormalities were mild, reversible, and normalized after dose adjustment or temporary discontinuation. Rare events, such as gastrointestinal discomfort or rash, occurred in <1% of patients and did not lead to treatment discontinuation. Overall, statins were well tolerated in CKD patients with cardiomyopathy, and although high-dose therapy slightly increased the incidence of adverse events, most were mild to moderate and reversible, with no severe adverse events observed.

**Table 8:** Adverse events associated with statin use.

Adverse event	Low-to-moderate dose (≤20 mg/d, n = 329)	High dose (>20 mg/d, n = 127)	Total (n = 456)	*P*-value
Muscle-related symptoms/CK elevation	14 (4.4%)	8 (6.3%)	23 (5.0%)	.36
Liver enzyme elevation (ALT/AST)	11 (3.3%)	7 (5.5%)	18 (3.9%)	.28
Gastrointestinal discomfort/rash	2 (0.6%)	2 (1.6%)	4 (0.9%)	.41
Rhabdomyolysis or severe events	0	0	0	–

ALT = alanine aminotransferase, AST = aspartate aminotransferase, CK = creatine kinase.

## 10. Discussion

This retrospective analysis of 765 patients with CKD combined with cardiomyopathy demonstrates that regular statin therapy is significantly associated with a reduced risk of MACE, and this protective effect exhibits a clear dose-dependent relationship. Patients receiving high-dose statins (>20 mg/day atorvastatin-equivalent) had a significantly lower incidence of MACE compared with the low–moderate dose group (≤20 mg/day), and this association remained robust after multivariable adjustment. Subgroup analyses further indicated that the protective effect was consistent across different CKD stages and cardiomyopathy etiologies. Moreover, statins were generally well tolerated, with adverse events primarily consisting of mild and reversible elevations of liver enzymes or CK, and no severe events were observed, suggesting a favorable safety profile and clinical applicability in this specific population.

CKD patients represent a high-risk population for atherosclerosis and cardiovascular events, and the presence of cardiomyopathy further amplifies cardiovascular risk. The pathogenesis is multifactorial, involving chronic inflammation, dyslipidemia, oxidative stress, and the accumulation of uremic toxins.^[11,12]^ In this study, statin users exhibited a significantly lower risk of MACE compared with nonusers, and multivariable logistic regression confirmed that regular statin therapy was an independent protective factor against MACE. These findings are consistent with previous studies by Fularski, Paparodis, and colleagues,^[13–15]^ suggesting that statins confer cardiovascular protection not only through LDL-C reduction but also via endothelial function improvement, anti-inflammatory effects, and inhibition of myocardial fibrosis. Importantly, our study revealed a dose-dependent cardiovascular benefit of statins. The high-dose group demonstrated a significantly lower MACE risk than the low–moderate dose group, indicating that adequate statin dosing may yield more pronounced clinical benefits in CKD patients with cardiomyopathy. Prior studies have shown that high-intensity statins more effectively suppress LDL-C and C-reactive protein levels, reduce myocardial ischemia and inflammatory injury, and improve outcomes.^[16–18]^ In clinical practice, CKD patients are often treated with lower statin doses due to concerns regarding adverse events. Our findings suggest that, under careful monitoring of hepatic, renal, and muscle enzyme parameters, moderate-to-high-intensity statin therapy is feasible and safe in this population.

The attenuated protective effect of statins observed in patients with stage 5 CKD may be explained by several pathophysiological factors. Advanced CKD is frequently accompanied by chronic systemic inflammation, protein-energy wasting, vascular calcification, and irreversible myocardial remodeling. These conditions may limit the lipid-lowering and pleiotropic benefits of statins, thereby reducing their cardiovascular protective effect in this subgroup. Additionally, competing risks such as non-atherosclerotic cardiovascular mortality may further obscure potential benefits.

Subgroup analyses further confirmed the broad applicability of statin therapy. Across different CKD stages (3–5) and cardiomyopathy etiologies (ischemic vs nonischemic), regular statin use was consistently associated with lower MACE risk, and interaction tests were not statistically significant. This indicates that the cardiovascular protective effect of statins is maintained despite declining renal function and varying cardiomyopathy pathology, supporting their use in a wider CKD population. Particularly for nonischemic cardiomyopathy, statins may improve myocardial structure and function through antioxidative and antifibrotic mechanisms,^[19,20]^ suggesting benefits beyond atherosclerosis prevention. Regarding safety, adverse events were predominantly mild elevations in liver enzymes or CK, which resolved completely upon dose adjustment or temporary discontinuation. No cases of rhabdomyolysis or severe hepatic injury were observed. The incidence of adverse events did not differ significantly between the low-to-moderate and high-dose groups, indicating that statins are well tolerated in CKD patients with cardiomyopathy.

As an observational retrospective study, causal inference cannot be definitively established. Although multivariable adjustment and PSM were applied to minimize confounding, residual confounding from unmeasured factors such as medication adherence, socioeconomic status, or lifestyle factors cannot be completely excluded. Therefore, the findings should be interpreted as associative rather than causal.

In conclusion, regular statin therapy significantly reduces the risk of MACE in CKD patients with cardiomyopathy, with a clear dose-dependent effect. Moderate-to-high-intensity statin therapy demonstrates both efficacy and safety, and should be considered a key component of cardiovascular risk management in this high-risk population.

## Author contributions

**Conceptualization:** Wansheng Qiu, Wei Gao, Lingyan Li, Xiaoyan Guo, Dandan Liu, Weiwei Liu, Xiong Yang.

**Data curation:** Wansheng Qiu, Wei Gao, Lingyan Li, Xiaoyan Guo, Dandan Liu, Weiwei Liu, Xiong Yang.

**Formal analysis:** Wansheng Qiu, Wei Gao, Lingyan Li, Xiaoyan Guo, Dandan Liu, Weiwei Liu, Xiong Yang.

**Funding acquisition:** Lingyan Li, Dandan Liu, Xiong Yang.

**Investigation:** Xiaoyan Guo, Xiong Yang.

**Writing – original draft:** Wansheng Qiu, Wei Gao, Lingyan Li, Xiaoyan Guo, Xiong Yang.

**Writing – review & editing:** Wansheng Qiu, Wei Gao, Lingyan Li, Xiaoyan Guo, Dandan Liu, Xiong Yang.
